# Obstructive Jaundice Due to Hepatocarcinoma With Intraductal Growth. Report of a Successful Resection

**DOI:** 10.1155/1990/38987

**Published:** 1990

**Authors:** L. C. Jiménez, Ana Teruel, Susana Mezquita, J. Martínez, F. Colina

**Affiliations:** 1 Department of Surgery, Pathology Hospital de Pozoblanco Avda. de la Constitución s/n° Pozoblanco Córdoba Spain; 2 Department of Gastroenterology Hospital de Pozoblanco Avda. de la Constitución s/n° Pozoblanco Córdoba Spain; 3 Department of Surgery Hospital de Pozoblanco Avda. de la Constitución s/n° Pozoblanco Córdoba Spain; 4 Lucio del Valle, 17 Madrid 4° D 28003 Spain

## Abstract

We present a patient with hepatocellular carcinoma causing obstructive jaundice due to intraductal growth, diagnosed intraoperatively by cholangiography and histological examination, and radically treated by left lobectomy, extrahepatic biliary tract resection and Roux-en-Y hepaticojejunostomy. Survival after operation was 13 months. Other similar cases reported in the literature are reviewed.


*HPB Surgery*, 1990, Vol. 2, pp. 73–76
Reprints available directly from the publisher
Photocopying permitted by license only

© 1990 Harwood Academic Publishers GmbH
Printed in the United Kingdom

### OBSTRUCTIVE JAUNDICE DUE TO HEPATOCARCINOMA WITH INTRADUCTAL GROWTH. REPORT OF A SUCCESSFUL RESECTION

L.C. JIMÉNEZ, ANA TERUEL*, SUSANA MEZQUITA, J. MARTÍNEZ** and F. COLINA*

*Departments of Surgery, Pathology* and Gastroenterology** Department of Surgery, Hospital de Pozoblanco, Avda. de la Constitución s/nº, Pozoblanco (Córdoba). SPAIN.*

*(Received 13 April 1989)*

We present a patient with hepatocellular carcinoma causing obstructive jaundice due to intraductal growth, diagnosed intraoperatively by cholangiography and histological examination, and radically treated by left lobectomy, extrahepatic biliary tract resection and Roux-en-Y hepaticojejunostomy. Survival after operation was 13 months. Other similar cases reported in the literature are reviewed.

KEY WORDS: Liver neoplasms, icterogenic hepatocarcinoma; obstructive jaundice; liver resection.

### INTRODUCTION

Jaundice associated with hepatoma usually occurs in later stages as a result of an infiltrating tumor or of underlying cirrhosis, but rarely due to tumour growth, fragment migration or clots in the biliary tract[1]. Reviewing the literature, there are 13 reported icterogenic hepatocarcinoma cases treated by hepatic lobectomy (8 left and 5 right) and 2 by segmentectomy; blood clots being the cause of obstruction in 2 and extraluminal biliary compression in one[2,3]. Hepaticojejunostomy was carried out in 3 cases[3]. We report a case of hepatocarcinoma growing into the intra- and extrahepatic bile ducts, diagnosed intraoperatively and treated by left lobectomy, extrahepatic biliary tract resection and hepaticojejunostomy.

### CASE REPORT

A 59-year-old woman was admitted on January 10, 1987 with a ten-day history of listlessness and anorexia with 2 kg weight loss. Two-days before admission she suffered pain in the right upper quadrant radiating to the right shoulder, with jaundice and lightening stools but without vomiting, fever or chills. She had had an episode of similar colic pain four months before, that had remitted with anticholinergics. She drank no alcohol. On physical examination she was alert, slightly obese, afebrile and jaundiced without signs of cirrhosis. The lungs and heart were normal. The abdomen was soft, moderately tender in the right upper quadrant

Correspondence to: L. C. Jiménez Romero MD C/ Lucio del Valle, 17, 4º D 28003–MADRID (SPAIN)

without rebound tenderness. A nontender liver was felt 1 finger breadth below the right costal margin. There was no ascitis nor peripheral oedema.

Examination of the blood revealed total bilirubin 6.7 mg/100 ml, direct bilirubin 4.6 mg/100 ml, alkaline phosphatase 1348 IU/1, SGOT 157 IU/1, SGPT 154 IU/1, GGT 2144 IU/1, negative AFP by RIA, negative Australia antigen and normal values of serum total protein and albumin.

Ultrasonography revealed an obstruction of the biliary confluence with dilatation in the intrahepatic ducts and the presence of a mass at that level, the gallbladder and extrahepatic bile duct being normal. The findings of the CT-scan were similar to ultrasonography, and celiac arteriography was normal. With these features a preoperative diagnosis of tumor of the biliary confluence was made.

Subcostal laparotomy was performed on January, 1987. No tumor was found on the surface of the cirrhosis free liver. The gallbladder and common bile duct were normal, and the upper level of the common hepatic duct and intrahepatic bile ducts were enormously dilated because of a non adherent neoplastic mass that occluded the lumen (Figure 1). The common hepatic duct was opened and a tumoral fragment removed for frozen section examination that revealed an hepatocarcinoma. The gallbladder, regional lymph nodes, common hepatic duct, hepatic bifurcation and left hepatic lobe (II, III and IV segments) were removed in an attempt to carry out a curative resection. The right hepatic duct was macroscopically free of tumor and an end to side right hepaticojejunostomy Roux-en-Y was performed.

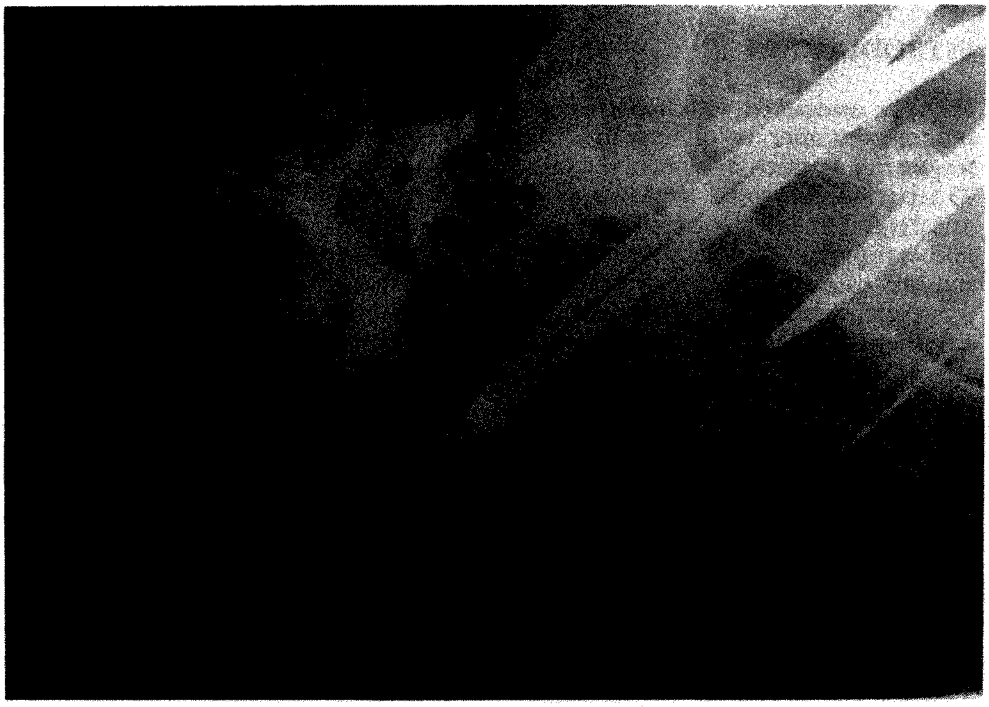

**Figure 1** Peroperative cholangiogram showing the marked dilatation of the left intrahepatic biliary tract because of the vegetant tumoral masses (arrows) that attain and occlude the common hepatic duct.

Macroscopically, the specimen of left hepatic lobectomy weighed 750 g and was 17 by 10 by 4 cm. The intrahepatic ducts of large to small size were markedly dilated and occluded by a whitish neoplastic tumor, but did not adhere to the wall of the ducts. Histologically, the tumor was moderately differentiated with predominantly intrabiliary growth, areas of necrosis and severe atypia (Figure 2). The extrahepatic biliary duct and lymph nodes removed were free of tumor. The postoperative course was uneventful, and the patient was discharged asymptomatic on the 15th day.

Until the twelfth month after operation the patient only suffered from slight dyspepsia with normal laboratory test results (including AFP and CEA). Hepatobiliary scintigraphy with $^{99}Tc^{m}$-HIDA performed 3 months after operation and ultrasonography at 3, 6 and 9 months were also normal. However, 12 months after discharge, the patient was readmitted because of an enormous and painful tumor located in the left upper quadrant. The ultrasonography and CT-scan disclosed a mass in the left upper quadrant bound to right hepatic lobe with displacement of the aorta, spleen, stomach and left kidney. The patient developed severe gastric bleeding, verified by endoscopy, because of penetration of the tumor through the gastric wall, dying 13 months after surgery. An autopsy was not performed.

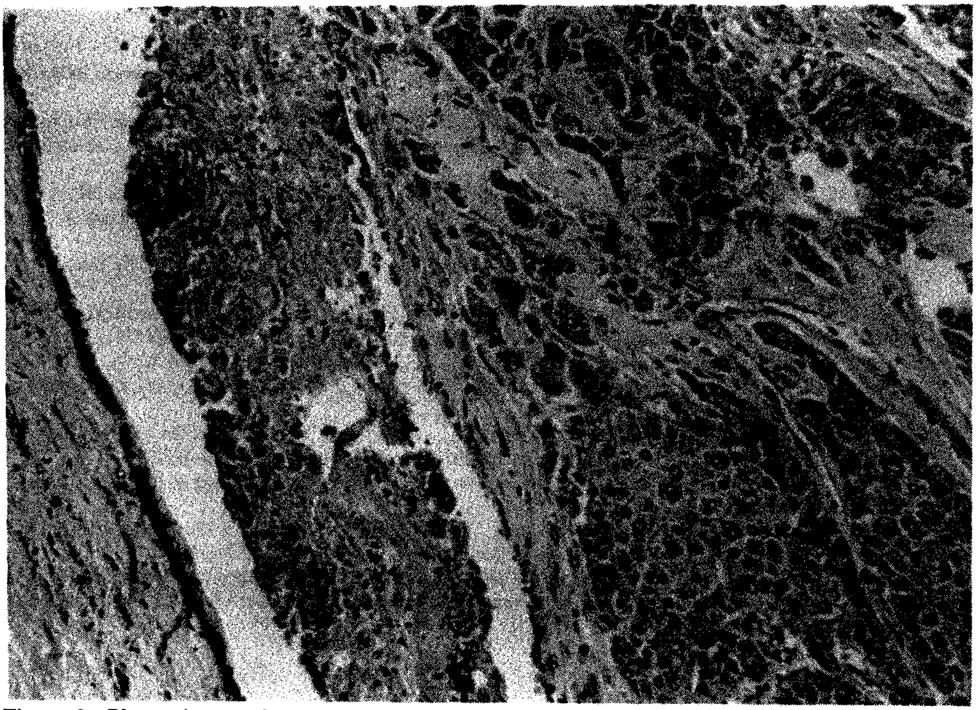

**Figure 2** Photomicrograph of hepatocellular carcinoma within a large intrahepatic duct (H & E, ×100).

### DISCUSSION

In large series the cholestatic type of hepatoma has a reported incidence of 1.2–1.5 per cent[4,5], but in a clinicopathologic study such incidence was 9 per cent[6].

In our presented case, the negative AFP, no previous alcohol consumption, the absence of liver mass visible by ultrasonography and CT-scan and the normality of celiac arteriography brought us, as others[1,7], to an erroneous preoperative diagnosis of bile duct tumor. With the ultrasonography, CT-scan, PTC and ERCP it is possible to detect the level of biliary obstruction, but the definitive diagnosis of this rare hepatoma will be feasible only by an histological study[6,8], intraoperative in our case.

At operation, frequently the liver tumor is not visible on the surface and too small to be detected by palpation[4,9], as was ours. Although good results have been reported in isolated cases with palliative procedures[1,9], the best treatment, when possible, will be a curative hepatic resection[4,10]. Our own case was radically treated by resection of the gallbladder, common hepatic duct and left hepatic lobe, including a regional lymphadenectomy with the aim of cure. Although the patient presented with a recurrence, twelve months later, the palliation obtained was worthwhile as in other resected cases[3,10]. An exception is the patient referred to by Eyskens *et al.*[2], that was alive four years after operation, perhaps owing to the better pronostic of fibrolamellar hepatocarcinoma.

In spite of the recorded recurrence in the icterogenic hepatocarcinoma, the survival of more than one year is possible with resection[3,10].

### *References*

1. Kuronayagi, Y., Sawada, M., Hidemura, R., Aoki, S., Kato, H., (1977) Common bile duct obstruction by hepatoma. *Am J Surg* **133**: 233–235.
2. Eiskens, E., Van der Stighelen, Y., Bourgeois. (1987) Icterogenic hepatocellular carcinoma and polygonal cell carcinoma with fibrous stroma (Fibrolamellar hepatocarcinoma). *Acta Chir Belg* **87**: 19--25.
3. Lee, K.C., Sakai, K., Kinoshita, H., Hirohashi, K., Tsuji, Y., Kubo, S., Iwasa, R. (1988) Resection of hepatocellular carcinoma with obstructive jaundice caused by compression of the common hepatic duct. *J Surg Oncol* **39**: 201–205.
4. Lin, T.Y., Chen, K.M., Chen, Y.R., Lin, W.S.J., Wang, T.H., Sung, J.L. (1975) Icteric type hepatoma. *Med Chir Dig* **4** 267–270.
5. Ihde, D.C., Sherlock, P., Winawer, S.J., Fortner, J.G., (1974) Clinical manifestations of hepatoma. Review of six years experience at a cancer hospital. *Am J Med* **56**: 83–91.
6. Kojiro, M., Kawabata, K., Kawano, Y., Shirai, F., Takemoto, N., Nakashima, T. (1982) Hepatocellular carcinoma presenting as intrabile duct tumor growth. A clinicopathologic study of 24 cases. *Cancer* **49**: 2144–2147.
7. Afroudakis, A., Bhuta, S.M., Ranganath, K.A., Kaplowitz, N. (1978) Obstructive jaundice caused by hepatocellular carcinoma. Report of three cases. *Dig Dis* **23**: 609–617.
8. Waldron, R.L., Kenny, G., Sorger, K. (1973) Liver-cell carcinoma presenting as bile-duct tumour. *Brit J Radiol* **46**: 195–197.
9. Capelle, P., Lichtenstein, H., Conte-Marti, J. (1972) Migration dans les voies biliaires de fragments d'hepatocarcinome. A propos de 3 cas. *Sem Hôp Paris* **48**: 177–182.
10. Tsuzuki, T., Ogata, Y., Iida, S., Kasajima, M., Takahashi, S. (1979) Hepatoma with obstructive jaundice due to the migration of a tumour mass in the biliary tract: Report of a successful resection. *Surgery* **85**: 593–598.

*(Accepted by S. Bengmark 17 May 1989)*

